# Abundant water from primordial supernovae at cosmic dawn

**DOI:** 10.1038/s41550-025-02479-w

**Published:** 2025-03-03

**Authors:** D. J. Whalen, M. A. Latif, C. Jessop

**Affiliations:** 1 Institute of Cosmology and Gravitation, Portsmouth University, Portsmouth, UK; 2 Physics Department, College of Science, United Arab Emirates University, Al-Ain, UAE

**Keywords:** Early universe, Computational astrophysics

## Abstract

Primordial (or population III) supernovae were the first nucleosynthetic engines in the Universe, and they forged the heavy elements required for the later formation of planets and life. Water, in particular, is thought to be crucial to the cosmic origins of life as we understand it, and recent models have shown that water can form in low-metallicity gas like that present at high redshifts. Here we present numerical simulations that show that the first water in the Universe formed in population III core-collapse and pair-instability supernovae at redshifts *z* ≈ 20. The primary sites of water production in these remnants are dense molecular cloud cores, which in some cases were enriched with primordial water to mass fractions that were only a factor of a few below those in the Solar System today. These dense, dusty cores are also probable candidates for protoplanetary disk formation. Besides revealing that a primary ingredient for life was already in place in the Universe 100–200 Myr after the Big Bang, our simulations show that water was probably a key constituent of the first galaxies.

## Abstract (web summary)

State-of-the-art computer simulations show that the first water in the Universe formed in primordial supernova remnants 100 Myr after the Big Bang, enriching future sites of planet formation to levels that were nearly those in the Solar System today.

## Main

**Fig. 1:**
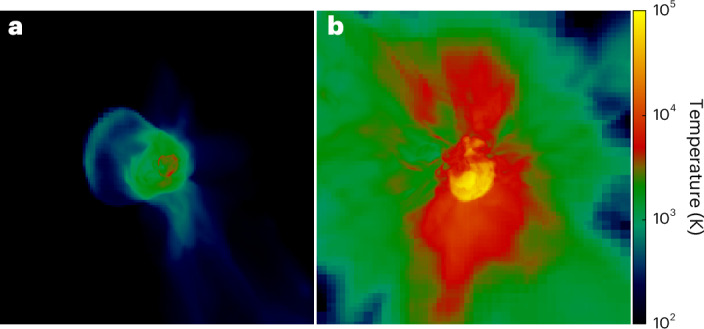
Primordial supernova explosions. **a**,**b**, The 13 *M*_⊙_ CC supernova in the 1.1 × 10^6^ *M*_⊙_ halo 1.2 Myr after the explosion (**a**) and the 200 *M*_⊙_ PI supernova in the 2.2 × 10^7^ *M*_⊙_ halo 0.7 Myr after the explosion (**b**) (images are 1 kpc on a side). The relic H ii regions of the stars are visible as the 2,000–10,000 K gas, and the CC and PI ejecta are visible as the 10^4^ and 10^5^ K shocked gas with radii of ~50 and 100 pc, respectively. At the end of the simulations, both supernova remnants are still trapped within their respective H ii regions.

We modelled the explosions of 13 *M*_⊙_ (where *M*_⊙_ is the solar mass) and 200 *M*_⊙_ population III (Pop III) stars^1–3^ with the Enzo adaptive mesh refinement code^4^. A 13 *M*_⊙_ star forms when a cosmological halo grows to 1.1 × 10^6^ *M*_⊙_ at *z* = 22.2. It lives for 12.2 Myr and then explodes as a core-collapse (CC) supernova with an energy of 10^51^ erg, ejecting 0.784 *M*_⊙_ of metals with 0.051 *M*_⊙_ of oxygen. In the second simulation, the 200 *M*_⊙_ star forms in a 2.2 × 10^7^ *M*_⊙_ halo at *z* = 17.8. It lives for 2.6 Myr and then explodes as a pair-instability (PI) supernova with an energy of 2.8 × 10^52^ erg, ejecting 113 *M*_⊙_ of metals with 55 *M*_⊙_ of oxygen^5–8^. Ionizing ultraviolet (UV) flux from both stars creates anisotropic H ii regions with final radii of about 150 and 500 pc, respectively^9–11^. Neither ionization front breaks out of its halo, so the explosions occur in trapped H ii regions^12^ with somewhat higher internal densities of ~1 cm^−3^. After the stars explode, H_2_, a key ingredient in water formation^13^, rapidly forms throughout their H ii regions because it cools faster than it recombines. As the supernova shock sweeps up gas in the halo, it cools, first by bremsstrahlung emission and then by collisional excitation and ionization of H and He (refs. ^14,15^). The two supernovae are shown in Fig. 1. The relic H ii regions are visible as the 2,000 to 10,000 K gas, and the CC and PI ejecta are visible as the 10^4^ and 10^5^ K shocked gas with radii of ~50 and 100 pc, respectively. At the end of the simulations, both supernova remnants are still trapped within their H ii regions.

## Water synthesis

**Fig. 2:**
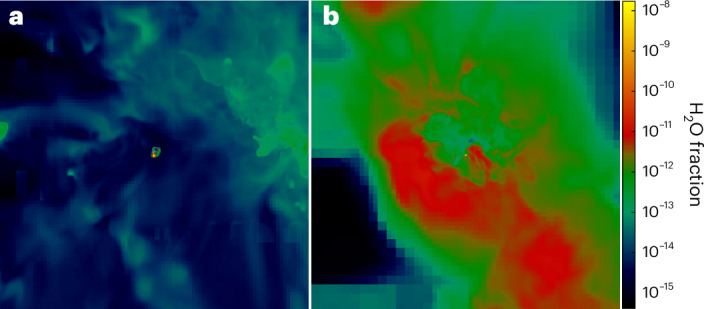
Water vapour in primordial haloes. **a**,**b**, Images simulated at 1 kpc distance of water vapour in the 13 *M*_⊙_ CC supernova at 90 Myr after the explosion (**a**) and the 200 *M*_⊙_ PI supernova at 3 Myr after the explosion (**b**). Mass fractions for diffuse water vapour in the haloes vary from 10^−14^ to 10^−12^ in the CC supernova and 10^−12^ to 10^−10^ in the PI supernova. Dense clumps with much higher water masses are visible as the yellow specks in the centres of both images.

**Fig. 3:**
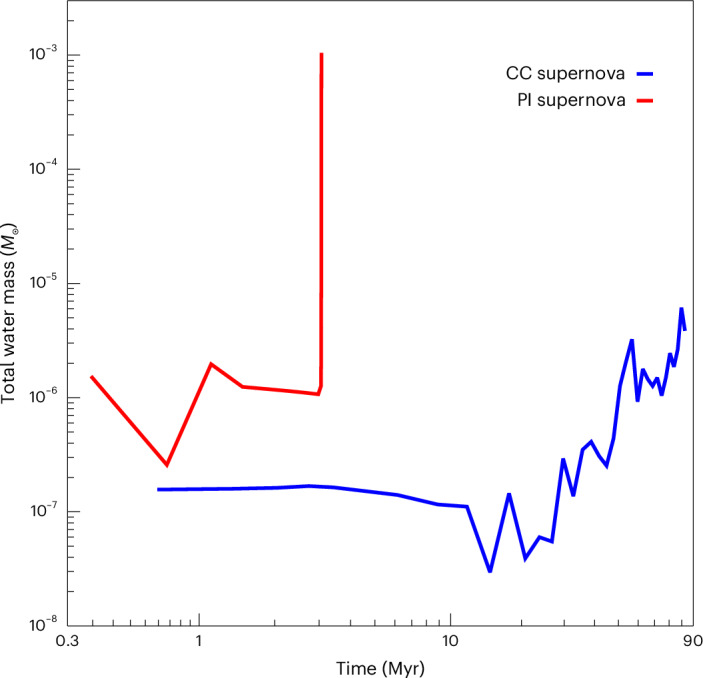
Supernova water masses. Total water masses in the CC (blue) and PI (red) supernovae as a function of time since the explosion. The total water masses are dominated by synthesis in dense cloud cores in their respective haloes at late times. Water formation rises sharply at earlier times in the PI supernova core because the cooling and collapse timescales are shorter at its higher metallicities.

As the supernovae expand and cool, oxygen from the ejecta reacts with H and H_2_ to form water in the halo, and H_2_ also forms on dust grains. As shown in Fig. 2, diffuse water vapour later permeates both haloes with mass fractions of 10^−14^ to 10^−12^ in the CC supernova and 10^−12^ to 10^−10^ in the PI supernova. Although water forms throughout both haloes, its total masses remain small and grow slowly over most of the simulation time, as seen in Fig. 3. The small masses and slow growth are due to the relatively low densities in the expanding supernova remnants, in which the reactions that produce water have low rates. The water mass grows from 10^−8^ to 10^−7^ *M*_⊙_ in the CC supernova over the first 20 Myr and from 1 to 1.5 × 10^−6^ *M*_⊙_ in the PI supernova over the first 2–3 Myr. As shown in Fig. 2, the water mass fractions in the PI supernova on large scales are highest in the dense shell of gas that is swept up and chemically enriched by the expanding shock because densities, and thus H_2_O reaction rates, are greatest there.

**Fig. 4:**
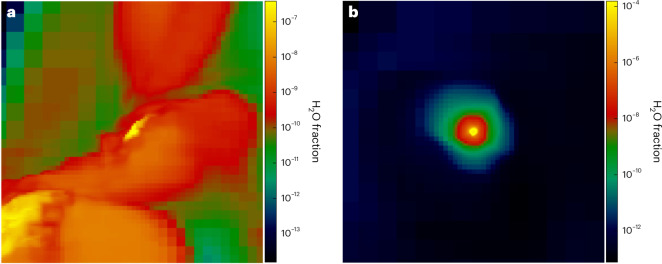
Water mass fractions in the dense cloud cores. **a**,**b**, Images of water mass fractions in the CC supernova core at 90 Myr (**a**) and the PI supernova core at 3 Myr (**b**). The images are 3.0 pc (**a**) and 0.1 pc (**b**) on a side.

However, the water masses then rise sharply by a few orders of magnitude in both haloes, from 10^−6^ *M*_⊙_ to 10^−3^ *M*_⊙_ at 3 Myr in the PI supernova and from 10^−8^ *M*_⊙_ to 10^−6^ *M*_⊙_ from 30–90 Myr in the CC supernova. This water forms almost entirely in two dense cloud cores, one in each halo, that were contaminated by metals from the explosions and then collapsed to high densities at which H_2_O reaction rates abruptly rise. As shown in Fig. 4, the water mass fractions reach 10^−4^ in the PI supernova fragment and 4 × 10^−7^ in the CC supernova core by the end of the runs, the latter of which is consistent with those in one-zone models at similar metallicities and densities^13^. The dominant sites of water production in primordial supernovae are, thus, dense, self-gravitating cores in the ejecta^16^, not the large volumes of diffuse gas enriched by water in the halo.

**Extended Data Fig. 1:**
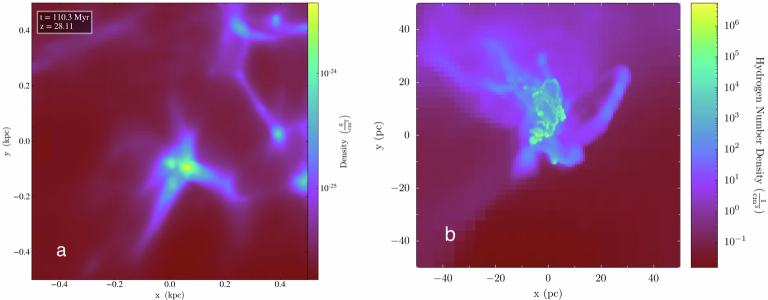
Clump formation in the CC SN halo. (a) The 2 × 10^5^ and 4.6 × 10^4^ solar mass halos at *z* = 28.11 (center) just before merging at *z* = 26.4 and later growing to 1.1 × 10^6^ solar masses and forming the 13 solar mass star at *z* = 22.2. (b) Zoom-in of the merged halo showing dense clumps created by turbulence during the encounter.

**Extended Data Fig. 2:**
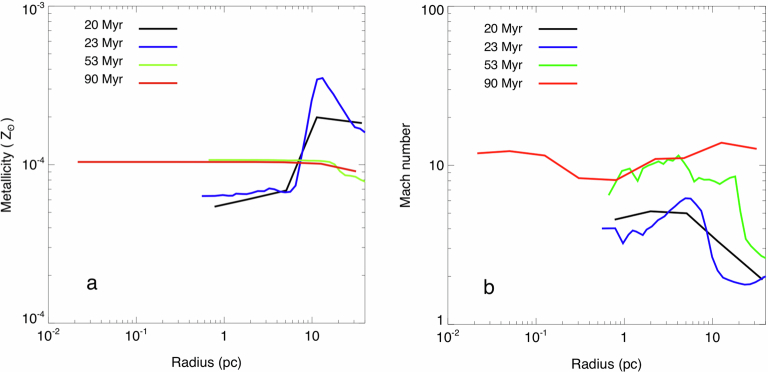
Enrichment of the clump by the CC SN. (**a**) Infusion of the clump with metals from the explosion at 20 Myr, 23 Myr, 53 Myr and 90 Myr. (**b**) Supersonic turbulence in the clump. At 20 Myr, the SN shock has just crashed into the clump and metals have begun to pile up in its outer layers at radii of 5 - 10 pc, with some reaching depths of ~ 0.7 pc. Relic transonic turbulence from the initial stages of collapse prior to the collision is visible in the low Mach numbers at 20 - 30 pc. The abrupt conversion of the bulk kinetic energy of inflow into random motions upon collision with the clump drives the supersonic turbulence at 5 - 10 pc, where the Mach numbers reach 5. At 23 Myr even more metals have piled up in the outer regions of the core and its internal metallicities have slightly risen. From 53 - 90 Myr the flows cause the entire core to become highly supersonically turbulent, and the metals that had previously accumulated in its outer regions now fully permeate it.

The CC supernova core formed before the explosion and is gradually enriched by it. As shown in Extended Data Fig. 1, turbulence in the wake of the merger of the two haloes at *z* = 26.4 that later host the 13 *M*_⊙_ star produces several gas clumps in its vicinity before its birth. One was only 30 pc away, and after surviving photoevaporation by the star, it collides with ejecta from the explosion 20 Myr later. As shown in Extended Data Fig. 2b, turbulence in the clump before the collision was transonic, with Mach numbers of ~2, as expected for self-gravitating cores in the initial stages of collapse. However, supernova flows then buffet the clump, which drives highly supersonic turbulence that gradually mixes it to metallicities *Z* ≈ 10^−4^ *Z*_⊙_ (where *Z*_⊙_ is the solar metallicity), as shown in Extended Data Fig. 2a. It collapses to a radius of ~0.1 pc at a mass of 1,627 *M*_⊙_, a central density of 2.4 × 10^8^ cm^−3^ and a total water mass of 10^−5^ *M*_⊙_ 90 Myr after the explosion.

**Extended Data Fig. 3:**
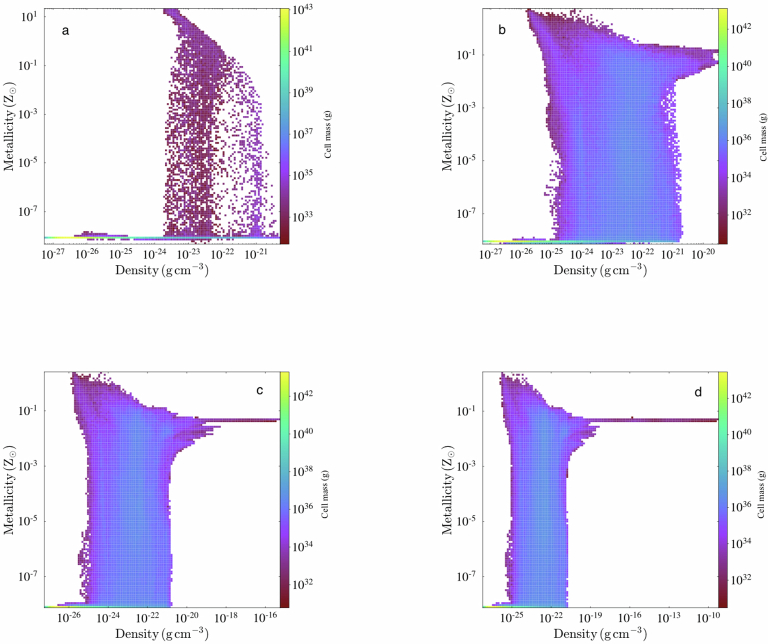
Emergence of the PI SN clump from *Z* = 0.04 Zsun gas. (**a**) The initial rapid enrichment of gas in the PI SN bubble 0.333 Myr after the explosion, in which the dense shell swept up by the expanding remnant (at ~ 10^−22^g cm^−3^) has already reached metallicities *Z* ~ 10^−2^ − 10^−3^ solar. (**b**) The appearance of a turbulent density fluctuation at ~ 10^−21^g cm^−3^ and *Z* ~ 0.1 − 0.01 solar at 1.86 Myr. (**c**) Initial collapse of the fluctuation to a dense core in *Z* = 0.04 solar gas at 3.05 Myr. (**d**) Runaway collapse of the core at 3.063 Myr.

**Extended Data Fig. 4:**
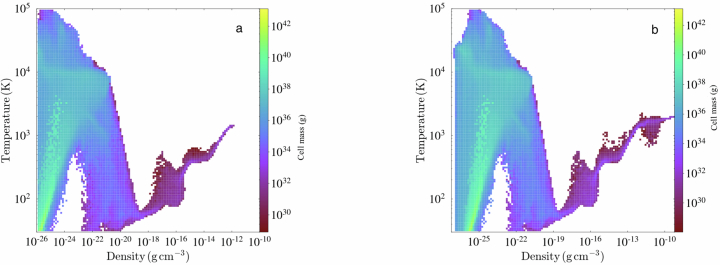
Onset of dust cooling in the PI SN clump. As central densities rise from 10^8^ − 10^11^cm^−3^, compressional heating increases gas temperatures from 400 - 1500 K as shown in (**a**). Dust cooling is then activated, and levels off central temperatures at 1200 K, cooling some of the gas down to 700 K as it collapses to ~ 10^14^ cm^−3^ by 7.7 kyr as shown in (**b**). Similar dips in temperature have been found in one-zone collapse tests with dust cooling. Not all the gas in the clump falls to a single temperature as in one-zone tests because of hydrodynamical effects in 3D such as turbulence, shocks and compressional heating, but the effects of dust cooling are evident in the dip in temperature in some of the gas at those densities. At present, we cannot follow the collapse of the dense core in the CC SNR to densities at which dust cooling becomes important.

The PI supernova core is created by the explosion. As shown in the phase diagrams in Extended Data Fig. 3, the hot PI supernova bubble^17,18^ promptly enriches surrounding gas to high (and even supersolar) metallicities at early times, as in previous cosmological simulations^19^. Hydrodynamical instabilities in the expanding bubble produce turbulent density fluctuations that form a compact clump of gas at *Z* = 0.04 *Z*_⊙_. It collapses to a radius of ~0.01 pc at a mass of 35 *M*_⊙_, a central density of 6.0 × 10^14^ cm^−3^ and a total water mass of 9 × 10^−3^ *M*_⊙_ 3 Myr after the explosion. At these densities, dust cooling becomes important in the core, as shown in the phase plots in Extended Data Fig. 4. This clump becomes self-gravitating at much earlier times than in the CC supernova because its higher metallicity results in faster cooling and collapse. In contrast, the CC supernova cloud core collapses on much longer timescales because of its much lower metallicities and only after first being mixed with external metals by supersonic turbulence. The CC supernova core forms and collapses on timescales like those at which metals have formed second-generation stars in previous cosmological simulations^12,20–22^. The PI supernova core will form such stars far earlier than in any simulation to date.

## Discussion and conclusion

Cloud cores enriched by metals from Pop III supernovae were probably the main sites of water formation in most primaeval haloes because similarities in explosion dynamics would have produced dense clumps across a wide range of energies, progenitor masses and halo masses. Conditions that favoured the formation of such cores, such as large mergers or explosions in trapped H ii regions, thus maximized the production of primordial water. Supernovae in compact H ii regions tend to form clumps because dynamical instabilities in the expanding ejecta form sooner and the shock stalls at earlier times in higher ambient densities. However, explosions can still form dense cores even if ionizing UV flux from the star breaks out of the halo and most of the baryons are lost to champagne flows because anisotropies in the H ii region can still trap radiation along some lines of sight.

We considered just a single star forming in each halo as the simplest case. Several stars may also form^23–27^. If so, several supernova explosions may occur and overlap in the halo. They may temporarily destroy water in low-density regions, but we expect that the dense cores where most water forms would survive ionizing UV and supernovae from other stars, just as the dense clump 30 pc from the 13 *M*_⊙_ star survived its radiation and explosion. Several explosions may produce more dense cores and, thus, more sites for water formation and concentration in the halo.

The highest redshift at which water has been found to date is *z* = 6.9 by detections of the p-H_2_O(2_1,1_ → 2_0,2_) and p-H_2_O(3_1,2_ → 2_1,2_) transition lines at 752 and 1,153 GHz with the Atacama Large Millimeter Array (ALMA)^28^. H_2_O(4_2,3_ → 4_1,4_) and H_2_O(3_3,0_ → 3_2,1_) lines at 2,264 and 2,196 GHz have also been discovered by ALMA in SPT 0346-52 at *z* = 5.656 (ref. ^29^). These lines, which are pumped by far-infrared emission from dust, would still be redshifted into ALMA bands at *z* ≳ 15. Gas and dust temperatures in our models exceed those that activate these transitions (~400 K), so primordial haloes emitted these lines. When the cores later form stars, dust can reprocess their radiation and populate metastable levels in H_2_O that produce maser emission at 22 GHz. With luminosities of 10^−3^ to 10^3^ *L*_⊙_ (where *L*_⊙_ is the solar luminosity), redshifted line emission from individual masers at *z* ≈ 20 would probably not be visible to the Square Kilometer Array or Next Generation Very Large Array today. However, a global population of these masers might have produced a cosmic line background at the end of the cosmic Dark Ages that could be found by these observatories in the coming decade. The same is true of far-infrared-pumped millimetre lines from haloes that could be detected by ALMA.

**Extended Data Fig. 5:**
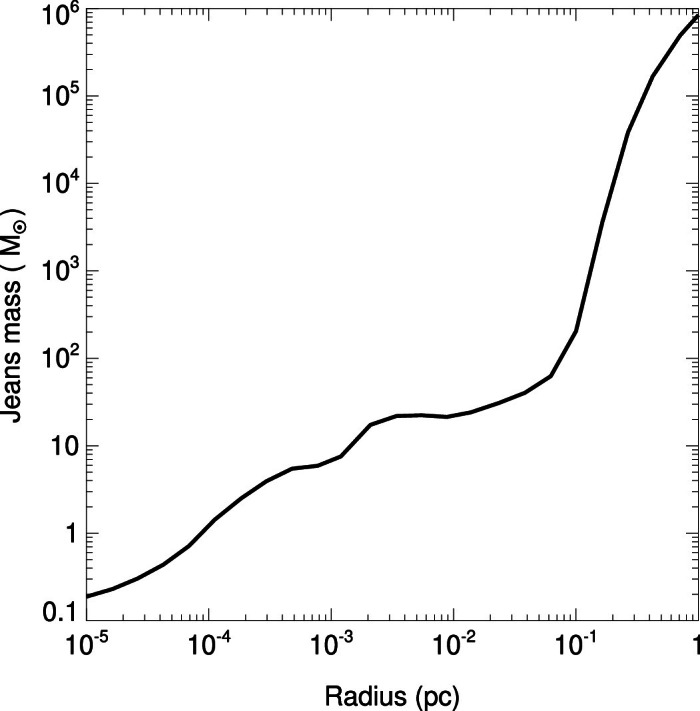
Jeans masses in the PI SN core. Gas at the center of the core can fragment on mass scales of ~ 1 solar mass.

Recent numerical simulations of exoplanet formation down to the lowest metallicities ever attempted indicate that both cores are probable sites of planet formation. Gas in the *Z* = 10^−4^ *Z*_⊙_ CC supernova clump could produce protoplanetary disks that fragment into Jupiter-mass planets^30^. The higher metal content of the *Z* = 0.04 *Z*_⊙_ PI supernova fragment could, in principle, lead to the formation of rocky planetesimals in protoplanetary disks with low-mass stars. This latter point is corroborated because Jeans masses fall to 1–2 *M*_⊙_ at the centre of the PI supernova core, as shown in Extended Data Fig. 5. We would not expect such planets to have much impact on exoplanet demographics today (except to extend its low-metallicity tail to smaller values) because Pop III stars were relatively sparse at *z* ≈ 15–20, but they could be detected as extinct worlds around ancient, metal-poor stars in the Galaxy in future exoplanet surveys^31^.

**Extended Data Fig. 6:**
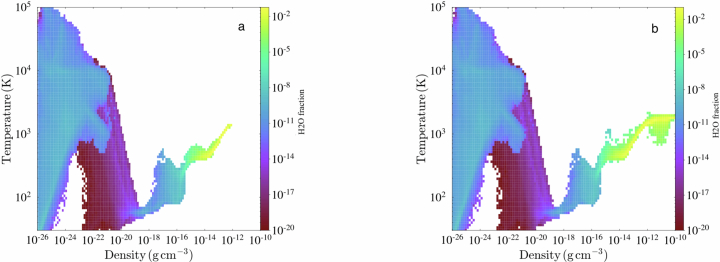
Water formation in the PI SN clump at the times in Extended Data Fig. 4. Compression and shock heating raise gas temperatures from 400 - 1500 K as central densities rise from 10^8^ − 10^11^ cm^−3^. **a**, Above n ~ 10^8^ cm^−3^, three-body formation of H_2_ rapidly molecularizes the core, which accelerates H_2_O formation via reactions Z10 - Z12. **b**, When dust cooling levels central temperatures off at 700 - 1200 K, cooling some of the gas down to 700 K as it collapses to ~ 10^14^cm^−3^, water reaches mass fractions of 10^−4^7.7 kyr later.

**Extended Data Fig. 7:**
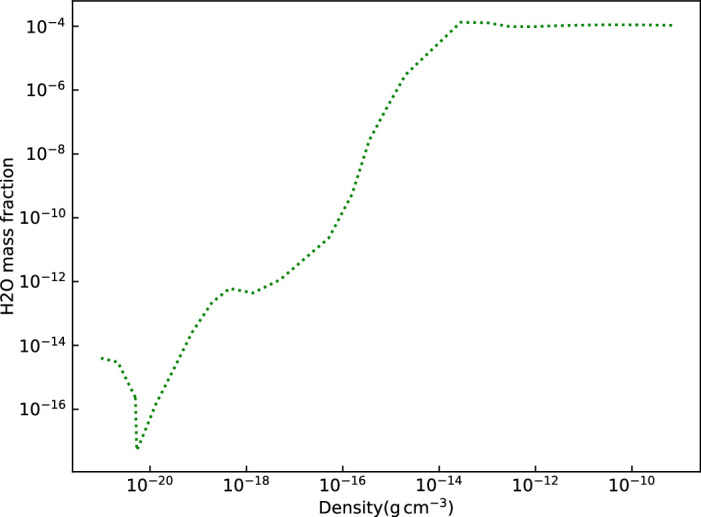
H_2_O mass fractions versus central density in the PI SN core. Central water mass fractions level of at ~ 10^−4^ above n ~ 10^10^, after three-body production of H_2_ has mostly molecularized the core.

**Extended Data Fig. 8:**
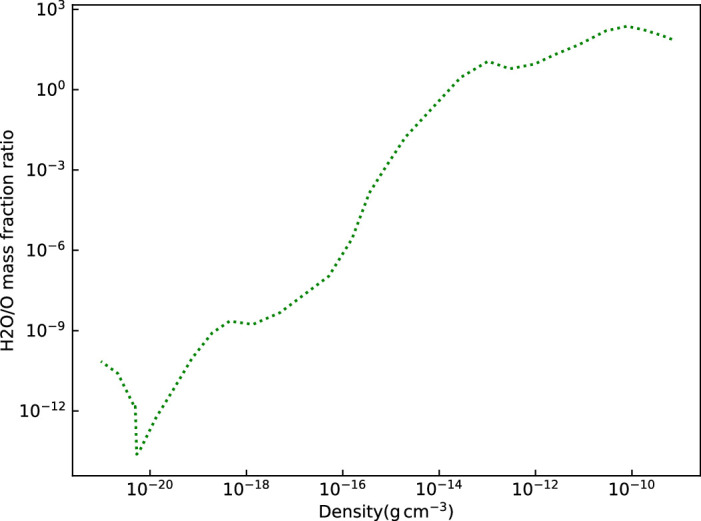
H_2_O/O mass fraction ratios in the PI SN core. Above n ~ 10^10^ when three-body production of H_2_ begins to fully molecularize the core, H_2_O/O mass fraction ratios exceed 1 and reach ~ 100, showing that most O depletes to H_2_O rather than O_2_.

As shown in Extended Data Figs. 6–8, our Enzo simulations show that these disks would have been heavily enriched by primordial water, to mass fractions that were 10–30 times greater than those in diffuse clouds in the Milky Way in the CC supernova core and to only a factor of a few lower than those in the Solar System today in the PI supernova core. The large H_2_O mass fractions and the potential for low-mass star formation in the PI supernova core raise the possibility of a habitable zone in the protoplanetary disk in which equilibrium temperatures allow water to exist in liquid form^32–34^. If planetesimals can form at *Z* = 0.04 *Z*_⊙_ in the disk, the planets into which they grow could harbour water.

Our simulations suggest that water was present in primordial galaxies because of its earlier formation in their constituent haloes^35–38^. Water mass fractions in diffuse supernova remnants taken up into these galaxies could reach 10^−10^, only an order of magnitude less than in the Milky Way today. Some of this water would have been photodissociated by massive, low-metallicity stars in these galaxies or destroyed by other chemical reactions as they reached higher metallicities at later times. However, rising dust fractions in early galaxies would also have shielded water from UV and mitigated its destruction to some degree. How much water survived the harsh radiation environments of the first galaxies remains to be determined.

## Methods

The Enzo cosmology code^4^ has an *N*-body particle-mesh scheme^39,40^ for evolving dark matter that is self-consistently coupled to hydrodynamics, non-equilibrium primordial gas chemistry and ionizing UV transport with the MORAY ray-tracing radiation code^41^. Our simulations use the piecewise parabolic method for hydrodynamics^42,43^ and the HLLC scheme for enhanced stability with strong shocks and rarefaction waves^44^. We use four energy bins in MORAY: H and He ionizing photons, H^−^ photodetachment photons and Lyman–Werner photons.

We implemented non-equilibrium water chemistry^22^ in Grackle^45^, with 49 reactions involving 18 primordial gas species (e^−^, H, H^+^, H_2_, H^−^, ${{\rm{H}}}_{2}^{+}$, HeH^+^, He, He^+^, He^2+^, D, D^+^, D^−^, HD, HD^+^, HeH^+^, D^−^ and HD^+^) and 40 reactions involving 19 metal and molecular species (C^+^, C, CH, CH_2_, CO^+^, CO, CO_2_, O^+^, O, OH^+^, OH, H_2_O^+^, H_2_O, H_3_O^+^, ${{\rm{O}}}_{2}^{+}$, O_2_, Si, SiO and SiO_2_)^46^. The 40 reactions are Z1 to Z35 and Z40 from Table 1 of ref. ^47^ and

$$\begin{array}{rcl}{{\rm{O}}}^{+}+{{\rm{e}}}^{-}&\to &{\rm{O}}+\gamma, \\ {\rm{Si}}+{{\rm{O}}}_{2}&\to &{\rm{SiO}}+{\rm{O}},\\ {\rm{Si}}+{\rm{OH}}&\to &{\rm{SiO}}+{\rm{H}},\\ {\rm{SiO}}+{\rm{OH}}&\to &{{\rm{SiO}}}_{2}+{\rm{H}},\end{array}$$

from Table 1 of ref. ^48^.

We evolve the reaction network with a fully implicit scheme. It includes updates to the gas energy due to heat from H_2_ formation and collisional excitation and ionization cooling by H and He, recombination cooling, bremsstrahlung cooling, and inverse Compton cooling by the cosmic microwave background at *T* > 8,000 K. We also include H_2_ and HD line cooling for *T* < 10,000 K, cooling due to fine structure emission by C^+^, C and O (ref. ^49^), and cooling by transitions between rotational levels in OH, H_2_O and CO, where the line rates are obtained from interpolations in precomputed tables^50–52^. Corrections to optically thin gas cooling by metal and molecule lines at high densities are also included^22^. Note that HD formation is evident in the relic H ii region of the PI supernova remnant in Extended Data Figs. 4 and 6, where it cools gas down to the temperature of the cosmic microwave background at that redshift.

UV photons can create and destroy H_2_O and its precursors, for example^53^,

$$\begin{array}{llll}{{\rm{H}}}_{2}{\rm{O}}+\gamma &\to &{{\rm{H}}}_{2}+{\rm{O}}&(h\nu > 9.5\,{\rm{eV}}),\\ {{\rm{H}}}_{2}{\rm{O}}+\gamma &\to &{\rm{H}}+{\rm{OH}}&(h\nu > 6.0\,{\rm{eV}}),\\ {\rm{OH}}+\gamma &\to &{\rm{O}}+{\rm{H}}&(h\nu > 6.4\,{\rm{eV}}).\end{array}$$

However, there are no other stars in the halo or its vicinity that could photodissociate water, so we did not include these reactions in our simulations. In principle, there could be diffuse UV emission from the remnant itself, but these fluxes would be much lower than those of nearby stars. Although these reactions would be required if other stars formed in the halo, there is enough dust in the cores to mitigate their effects to some degree. We did include photodissociation of H_2_ by 11.18 to 13.6 eV Lyman–Werner UV photons and photodetachment of H^−^ by continuum photons above 0.755 eV.

H_3_O^+^ formation by cosmic rays (CRs) is an important pathway to water formation in the Galaxy today. However, we expect CR densities in the primordial Universe to be much lower than in the Milky Way because Pop III star formation was relatively sparse. Consequently, supernovae, which produce CRs through first- and second-order Fermi acceleration in the shock, had low event rates, so *z* ≈ 20 was too early for a strong CR background to have arisen. We, therefore, excluded H_2_O formation through the H_3_O^+^ channel in our simulations. However, the PI and CC supernovae themselves produced some CRs, so we somewhat underestimated H_2_O mass fractions in the diffuse gas and dense cores.

Dust formation and destruction and gas cooling due to thermal emission from dust grains^22^ were also included in our simulations^48,54,55^. We included eight dust species: metallic silicon (Si), metallic iron (Fe), forsterite (Mg_2_SiO_4_), enstatite (MgSiO_3_), amorphous carbon (C), silica (SiO_2_), magnesia (MgO) and troilite (FeS). Our model includes the ten chemical reactions that create these species from Table 2 of ref. ^48^ along with

$$\begin{aligned}{\rm{CO}}+2{{\rm{H}}}_{2}&\to{{\rm{CH}}}_{3}{\rm{OH}},\\ {{\rm{H}}}_{2}{{\rm{O}}}_{({\rm{g}})}&\to{{\rm{H}}}_{2}{{\rm{O}}}_{({\rm{s}})},\end{aligned}$$

where (g) and (s) refer to gas and solid phases, respectively. Five of the 12 reactions deplete water onto dust grains, whereas the rapid catalysis of H_2_ by dust grains promotes water formation^47^. Dust grains do not directly catalyse water formation at *z* ≈ 20 because the cosmic microwave background maintains them at temperatures of 60 K, which are too high for reactants to bind to their surfaces.

Our CC supernova dust yields, compositions and grain size distributions are taken from ref. ^22^, which are from ref. ^55^. The CC supernova grain size distribution varies as *r*^−3.5^, like that in the Milky Way today^56^. This distribution and our dust composition are shown in Figs. 3 and 2 of ref. ^22^, respectively. Our PI supernova yields and grain sizes were taken from ref. ^55^. Those authors solved detailed nucleation models to obtain dust grain radii, and they included destruction due to thermal sputtering in the reverse shock of the supernova remnant. Because the strength of the reverse shock is determined in part by ambient densities, their dust yields are parametrized by the H ii region density. We adopted grain yields for 13 *M*_⊙_ and 200 *M*_⊙_ CC and PI supernova explosions in ambient densities of 1 cm^−3^ from ref. ^55^, as these densities were closest to those in their respective H ii regions in Enzo. These dust mass fractions are relatively low because the nucleation models predict that up to 90% of the dust originally formed in the explosion will be destroyed by the reverse shock at these local densities. However, these and other calculations of grain growth in supernovae^57^ are based on one-dimensional Lagrangian explosion models that exclude hydrodynamical instabilities in three dimensions, which can lead to the formation of dense clumps like the two studied here. Hydrodynamical studies have since shown that clumping in actual reverse shocks in three dimensions can shield dust from thermal sputtering^58^. Consequently, our dust mass fractions should be taken as (possibly severe) lower limits.

We initialized our CC supernova and PI supernova simulations in 1*h*^−1^ Mpc and 1.5*h*^−1^ Mpc boxes at *z* = 200 with cosmological initial conditions generated with MUSIC^59^ from the second-year Planck best-fitting lowP + lensing + BAO + JLA + H0 cosmological parameters: *Ω*_M_ = 0.3089, *Ω*_*Λ*_ = 0.691, *Ω*_b_ = 0.0486, *σ*_8_ = 0.816, *h*^−1^ = 0.677 and *n* = 0.967 (ref. ^60^). We first performed low-resolution (256^3^) unigrid dark matter-only runs to select haloes for the two stars. In the CC supernova run, we then centred two nested grids on the halo that spanned 10% of the top grid and evolved it with full baryonic physics down to 20 levels of refinement and four zones per Jeans length for a maximum resolution of 2,063 au. In the PI supernova run, we centred three nested grids on the halo. These spanned 10% of the top grid, and we evolved it with full baryonic physics with up to 28 levels of refinement and 16 zones per Jeans length to achieve a maximum resolution of 2.1 au. In the PI supernova run, we included a Lyman–Werner UV background of 100*J*_21_, where *J*_21_ = 10^−21^ erg^−1^ s^−1^ Hz^−1^ sr^−1^. This delayed star formation until the halo had grown to a little above 10^7^ *M*_⊙_.

We chose 13 *M*_⊙_ and 200 *M*_⊙_ progenitors because they lie near the centre of the mass ranges expected for these events, 8–20 *M*_⊙_ for most CC supernovae and 140–260 *M*_⊙_ for PI supernovae. The energy of the CC supernova, 10^51^ erg, also lies near the middle of those observed for most of these events, 0.6 to 2.4 × 10^51^ erg, whereas the PI supernova energy is an emergent feature of thermonuclear burning of O and Si in the stellar evolution models that produce the explosion. Elemental abundances for the CC supernova are shown in Fig. 1 of ref. ^22^. The PI supernova abundances exhibit the usual ‘odd–even’ effect, in which even-numbered nuclei are preferentially synthesized over odd-numbered nuclei by 1–2 dex. Energies and nucleosynthetic yields for both supernovae are taken from precomputed databases^2,61^.

### Water formation pathways during collapse

The primary H_2_O formation channels during collapse of the PI supernova core are reactions Z10 to Z12:

$$\begin{array}{llll}{\rm{Z}}10{:}&{\rm{O}}+{\rm{H}}&\to &{\rm{OH}}+\upgamma, \\ {\rm{Z}}11{:}&{\rm{O}}+{{\rm{H}}}_{2}&\to &{\rm{H}}+{\rm{OH}},\\ {\rm{Z}}12{:}&{\rm{OH}}+{{\rm{H}}}_{2}&\to &{{\rm{H}}}_{2}{\rm{O}}+{\rm{H}}.\end{array}$$

Z10 is more important in the early stages of the collapse, but Z11 takes over OH formation at *n* ≈ 10^8^ to 10^10^ cm^−3^ when the three-body formation of H_2_ begins to molecularize the core. At *Z* = 10^−^3 *Z*_⊙_, in previous idealized one-zone models of collapse^47^ most of the O goes into O_2_ during collapse through reaction Z19,

$${\rm{Z}}19{:}\quad{\rm{O}}+{\rm{OH}}\to{{\rm{O}}}_{2}+{\rm{H}}$$

because temperatures in those models stay below 300 K. However, this is not so in our PI supernova core, where compressional and shock heating keeps gas above 300 K (and closer to 1,000 K most of the time), despite its high metallicity, 0.04 *Z*_⊙_, as shown in Extended Data Fig. 6. There and in Extended Data Fig. 7, it can be seen that the central water mass fractions level off at ~10^−4^ at densities above 10^10^ cm^−3^, after three-body production has mostly molecularized the core. We also show the evolution of the ratio of H_2_O/O mass fractions versus central density during collapse in Extended Data Fig. 8, where it is seen that H_2_O formation dominates O_2_ formation above *n* ≈ 10^10^ cm^−3^. Z10 to Z12 also dominate H_2_O formation in the CC supernova core. Although we can follow its collapse only to central densities *n* ≈ 10^8^ cm^−3^, most of the O goes into H_2_O instead of O_2_ because of the lower metallicity, ~10^−4^ *Z*_⊙_, consistent with Fig. 5c of ref. ^47^.

## Data Availability

The Enzo parameter files and initial conditions files generated by MUSIC that are required to perform the simulations are available via Zenodo at 10.5281/zenodo.5853118 (ref. ^62^). The MUSIC input files required to generate the initial conditions are available at https://sites.google.com/site/latifmaastro/ics.

## Code availability

The code used to produce our cosmological simulations, Enzo 2.6, can be found at https://bitbucket.org/enzo/enzo-dev/tree/enzo-2.6.1.
